# The Role of Lifestyle Factors in Multiple Sclerosis: An Integrative Perspective

**DOI:** 10.3390/brainsci16020224

**Published:** 2026-02-13

**Authors:** Roberta Lanzillo, Marinella Clerico, Saverio Stranges

**Affiliations:** 1 Department of Neurosciences, Reproductive and Odontostomatological Sciences, Federico II University of Naples, 80138 Naples, Italy; 2 MS Center, Federico II University Hospital, 80131 Naples, Italy; 3 Department of Clinical and Biological Sciences, University of Turin, 10043 Orbassano, Italy; marinella.clerico@unito.it; 4 San Luigi Gonzaga University Hospital, 10043 Orbassano, Italy; 5 Department of Epidemiology & Biostatistics, Western University, London, ON N6G 2M1, Canada; saverio.stranges@uwo.ca; 6 Department of Family Medicine, Western University, London, ON N6G 2M1, Canada; 7 Department of Medicine, Western University, London, ON N6G 2M1, Canada; 8 Department of Clinical Medicine and Surgery, University of Naples Federico II, 80131 Naples, Italy

**Keywords:** lifestyle, multiple sclerosis, nutrition, sleep, risk factors

## Abstract

Multiple sclerosis (MS) is a chronic, immune-mediated neurological disorder characterized by inflammation, demyelination, and progressive neurodegeneration. While genetic susceptibility contributes to disease risk, a growing body of evidence highlights the crucial role of modifiable lifestyle factors in influencing MS onset, disease activity, progression, and overall quality of life. In this narrative review, we explored the relevant literature from commonly used datasets (PubMed, Scopus, Google Scholar), using search terms such as “Lifestyle and Multiple Sclerosis”, “Diet and Multiple Sclerosis”, “Sleep and Multiple Sclerosis”, “Alcohol consumption and Multiple Sclerosis”, and “Physical Activity and Multiple Sclerosis”. Obesity, particularly during adolescence, has emerged as a significant risk factor for MS, acting through immunometabolic mechanisms such as chronic low-grade inflammation, insulin resistance, and dysregulated adipokine signaling. Sleep disturbances are increasingly recognized as contributors to neuroinflammation and cognitive dysfunction, potentially mediated by impaired glymphatic clearance. Smoking is consistently associated with accelerated disability progression, while alcohol consumption shows dose-dependent effects, with excessive intake negatively impacting sleep and glymphatic function. Overall, lifestyle factors converge on shared biological pathways involving immune regulation, metabolic health, vascular integrity, and glymphatic function. Integrating evidence-based lifestyle counseling with disease-modifying therapies may represent a complementary strategy to optimize long-term outcomes in people with MS, while highlighting key areas for future translational and clinical research.

## 1. Introduction

Multiple sclerosis is a chronic demyelinating disorder of the central nervous system characterized by inflammatory and neurodegenerative processes [1]. While genetics play a key role in susceptibility, environmental and lifestyle-related factors are increasingly recognized as crucial contributors to disease risk and progression. Notably, global MS prevalence has doubled or tripled in countries such as Argentina, China, Egypt, and Germany over the past decade, possibly reflecting shifts in population aging, nutrition, physical activity, and other lifestyle domains, as well as in environmental factors and prolonged survival [2]. Understanding these modifiable contributors provides clinicians and researchers with opportunities for preventive strategies and personalized interventions.

## 2. Methods: Literature Search Strategy

This purpose of this narrative review was to explore relevant literature from reputable and commonly used datasets (PubMed, Scopus, Google Scholar) up to 2025, using search terms such as “Lifestyle and Multiple Sclerosis”, “Diet and Multiple Sclerosis”, “Sleep and Multiple Sclerosis”, “Alcohol consumption and Multiple Sclerosis”, and “Physical Activity and Multiple Sclerosis”. As this was a descriptive narrative review, no formal risk-of-bias assessment or quality appraisal was conducted, consistent with the descriptive and exploratory nature of this review.

## 3. Diet, Obesity, and MS Risk

Evidence linking dietary factors to multiple sclerosis risk and disease activity was predominantly gathered from observational cohort and cross-sectional studies, with more limited and often inconsistent data from randomized controlled trials. Observational analyses have reported that higher overall diet quality and Mediterranean-style dietary patterns are associated with lower disability levels and reduced symptom burden in people with MS, whereas Western-style dietary patterns correlate with worse metabolic and inflammatory profiles [3,4,5].

With regard to specific nutrients, vitamin D is the most extensively studied dietary-related factor in MS. Large observational studies consistently show that lower serum vitamin D levels are associated with increased relapse risk, MRI activity, and disability progression [6]. However, randomized controlled trials of vitamin D supplementation have produced mixed or neutral clinical results, with limited or no significant effects on relapse rates or disability progression despite measurable immunological changes [7,8]. Even more recently in a cohort of individuals following a clinically isolated syndrome (CIS), supplementation with vitamin D did not significantly reduce subsequent multiple sclerosis disease activity, indicating a lack of effect on early inflammatory outcomes [9], while in the randomized D-Lay MS trial, high-dose vitamin D supplementation in patients with CIS typical of multiple sclerosis did not demonstrate a reduction in disease activity compared with placebo, suggesting that even intensified vitamin D dosing does not alter early disease progression [10]. These discrepancies suggest that vitamin D status may partly reflect disease risk or inflammatory state rather than representing a fully modifiable causal driver in all patients.

For omega-3 fatty acids and other nutritional supplements, mechanistic and experimental data support potential anti-inflammatory effects, but randomized clinical trials in MS have generally shown small, inconsistent, or null effects on clinical and MRI outcomes [11,12]. Systematic reviews of dietary interventions in MS likewise conclude that the overall quality of interventional evidence remains moderate to low and heterogeneous across study designs [5].

However, several studies have identified obesity, particularly during adolescence and early adulthood, as a significant modifiable risk factor for MS. Mokry LE and colleagues [13] demonstrated a strong causal link between elevated BMI and increased MS risk susceptibility, using Mendelian randomization methods. These findings highlight that the inflammatory and immunological consequences of central adiposity are stronger drivers of disease susceptibility and progression as compared to dietary habits.

Pediatric MS is a very interesting field in terms of studying dietary influences on MS. A notable contribution to the nutritional perspective in MS, with specific attention to pediatric populations, is provided by Mandato et al. [14]. In this comprehensive scoping review, the authors synthesize evidence on dietary habits, nutritional status, and potential dietary interventions in children and adolescents with MS, highlighting how factors such as obesity, vitamin intake—particularly vitamin D—and overall diet quality may influence the risk of onset, disease course, and metabolic comorbidities. The review underscores the need for multidisciplinary management and well-designed prospective studies in pediatric MS, where data remain limited as compared to adults. This work broadens and strengthens the evidence base for nutritional contributors to MS, supporting the importance of balanced dietary strategies from early life [14].

Current data do not identify any negative interactions between lifestyle interventions and DMTs. On the contrary, observational studies and guideline reviews suggest that lifestyle modifications—including Mediterranean-style diet, regular physical activity, stress reduction, and sleep optimization—may provide additive benefits to DMTs by improving symptom burden, reducing comorbidities, and creating a biologically favorable environment for treatment response. A recent trial on curcuma—a naturally occurring poly-phenolic phytochemical with potent anti-inflammatory and antioxidant properties—in subjects under treatment with IFN β-1a to test the effects of this combination therapy on clinical and MRI parameters of inflammation and neurodegeneration in relapsing MS (RMS), suggested that curcumin add-on might improve efficacy on radiological signs of inflammation in MS, while it did not seem to exert any neuroprotective effect as assessed by clinical and MRI parameters [15].

Taken together, these findings indicate that while dietary optimization and weight control are reasonable supportive strategies for overall metabolic and cardiovascular health, the strength of evidence differs substantially between observational associations and randomized interventional data, and this distinction should guide both clinical counseling and future research priorities.

## 4. Physical Activity: Prevention and Management

Physical activity has emerged as a key factor in both the prevention and symptomatic treatment of MS. In the EnvIMS study [16], individuals engaging in high levels of vigorous activity had a significantly lower risk of developing MS, independent of traditional risk factors. In established MS, regular physical activity improves aerobic capacity, mobility, fatigue, and quality of life.

Randomized controlled trials have highlighted the clinical impact of structured exercise programs. Langeskov–Christensen M and colleagues [17] reported that a 24-week supervised high-intensity aerobic regimen resulted in significantly fewer relapses and improved motor function. Additional research suggests that even in the absence of measurable changes in brain volume over short timeframes, exercise contributes to functional stability and mitigates symptom progression [18]. Systematic reviews including both observational studies and randomized clinical trials corroborate potential benefits of regular resistance and aerobic exercise in improving fatigue and quality of life among MS patients [18]. Despite this, many pwMS remain insufficiently active, highlighting the need for tailored, accessible, and motivating interventions such as aquatic therapy and balance/motor training. Greater attention should be paid to exercise dosing, adherence, and long-term outcomes in future studies. Exercise prescription across all MS phenotypes is best guided by the National MS Society’s consensus recommendations, which advise combining aerobic, resistance, and flexibility training at individualized intensities to reach approximately 150 min of weekly activity throughout the disease course [19].

## 5. Sleep and Glymphatic Function

The glymphatic system, a perivascular network responsible for clearing interstitial waste from the brain, plays an essential role in maintaining CNS homeostasis [20]. Disruptions in this system have been implicated in several neurodegenerative conditions, and emerging evidence suggests a similar relevance in MS. Sleep health is a key modulator of glymphatic activity; during deep sleep, CSF flow and interstitial clearance are optimized [21]. Epidemiological studies suggest insufficient sleep or poor quality sleep may represent an independent risk factor for multiple sclerosis (MS). The glymphatic system, active during slow-wave sleep, clears brain waste through perivascular astrocytic aquaporin-4 (AQP4 channels) [22]. The presence of antigens induces a transient, physiological lowering of glymphatic flux as a first step of an inflammatory response. A possible hypothesis linking infection with the Epstein–Barr virus, a well-identified causal step in MS, and the development of the disease is that mechanisms such as poor sleep or less functional AQP4 polymorphisms may sustain glymphatic flow reduction [23]. Such chronic glymphatic reduction would trigger a vicious circle in which the persistence of antigens and an inflammatory response maintain glymphatic dysfunction. In addition, viral proteins that persist in demyelinated plaques can depolarize AQP4, further restricting waste elimination and sustaining local inflammation. So, there is rising epidemiological evidence connecting poor sleep and MS risk, and the mechanistic findings show how disrupted sleep patterns and other glymphatic modulators heighten inflammatory signaling implicated in MS pathogenesis [24].

In pwMS, sleep disorders—including insomnia, fragmented sleep, and poor sleep efficiency—are highly prevalent and correlate with cognitive impairment, mood disorders, and fatigue [25].

Carotenuto et al. [26] demonstrated for the first time that glymphatic dysfunction is detectable in MS using diffusion-based MRI measures, showing that the diffusion-along-perivascular-space (DAPS) index is significantly reduced in both relapsing–remitting and progressive MS compared with healthy controls. Lower DAPS values were strongly associated with greater clinical disability, longer disease duration, increased lesion burden, and gray-matter atrophy, with the most pronounced impairment observed in progressive MS patients. Notably, the relationship between reduced glymphatic function and disease duration was most evident during the early years following onset, suggesting that glymphatic impairment may emerge early in the disease course and contribute to subsequent neurodegeneration. These findings position diffusion-based perivascular metrics as promising, though still experimental, biomarkers for studying glymphatic alterations in MS and their relationship to inflammatory and neurodegenerative pathology.

Shokri–Kojori E [27] demonstrated that even a single night of sleep deprivation leads to significant amyloid-beta accumulation. Other evidence further highlights how chronic sleep dysfunction affects glymphatic clearance through alterations in astrocytic AQP4 polarization and neuroinflammation [23,24]. Thus, strategies to improve sleep duration and quality, such as behavioral therapies, sleep hygiene education, and chronotherapy, may offer therapeutic value in MS beyond symptomatic relief. The critical role of sleep in MS is further amplified by the potential detrimental impact of poor sleep patterns on a wide range of health outcomes and chronic comorbidities including immune disorders, and cardiometabolic, neurodegenerative and psychiatric disease [28].

The convergence of lifestyle factors on glymphatic function represents a compelling unifying mechanism in MS pathogenesis, supported by converging experimental and translational evidence on sleep physiology, vascular pulsatility, and astrocytic AQP4-dependent clearance pathways [22,23]. Reduced cerebrospinal fluid–interstitial fluid exchange has been demonstrated in experimental and human studies under conditions such as sleep deprivation, vascular dysfunction, and alcohol exposure, and may contribute to waste accumulation, neuroinflammation, and axonal degeneration [22,23]. Sleep loss has been directly associated with impaired metabolite clearance and increased neurotoxic protein burden in the human brain [22,23], while alcohol shows dose-dependent effects, with low-dose exposure transiently enhancing and high-dose exposure impairing glymphatic function and AQP4 polarization [23]. Sedentary behavior and obesity are also linked to vascular and metabolic alterations that reduce arterial pulsatility and perivascular flow, which are the two major drivers of glymphatic transport [13,16]. As the glymphatic system is closely coupled with vascular pulsatility and cerebral perfusion, lifestyle habits that promote cardiovascular and sleep health are therefore biologically plausible contributors to preserved glymphatic efficacy, although direct MS-specific clinical validation remains limited [13,16,17]. This integrative model positions the glymphatic system as both a biomarker and a therapeutic target. Physical exercise, proper hydration, healthy sleep, and vascular-friendly diets all support optimal glymphatic function. Future research based on prospective studies and randomized clinical trials should explore the longitudinal impact of these interventions on imaging biomarkers of brain clearance and inflammation in pwMS.

## 6. Smoking and Alcohol: Impact on MS Progression

Smoking remains one of the most detrimental lifestyle factors in MS. Smokers with MS exhibit higher EDSS and MSSS scores, increased MRI lesion load, greater brain atrophy, and a faster transition to secondary progressive disease [29]. Studies also show that smoking exacerbates pro-inflammatory pathways while impairing immune surveillance, especially in the context of Epstein–Barr virus reactivation [30]. Importantly, smoking cessation significantly slows disease progression, making it a primary therapeutic recommendation for all pwMS [31].

Alcohol consumption presents a more complex picture. While moderate consumption may transiently enhance glymphatic clearance through nitric oxide-mediated vasodilation, excessive intake disrupts astrocytic function and impairs CSF flow [32]. Moreover, alcohol-related sleep disruption compounds glymphatic impairment [33]. As such, clinicians should encourage moderation and screen for problematic alcohol use, especially in patients with sleep or cognitive issues.

## 7. Importance of Adopting a Life Course Approach

Early pharmacological and lifestyle interventions are essential in chronic disease such as MS for promoting long-term well-being and quality of life, as well as reducing the risk of chronic comorbidities and accelerated progression [34]. Indeed, this approach recognizes that chronic disease is influenced by an accumulation of factors throughout a person’s life, starting from early development and extending into adulthood and old age. A life course approach emphasizes the importance of establishing healthy lifestyles early in life to mitigate long-term health risks. This is even more critical in the context of disabling long-lasting diseases such as MS, slowing down disease progression, reducing the risk of chronic comorbidities, and preserving quality of life [35]. Timely implementation of routine assessment and early effective interventions to positively influence disease prognosis as well as ensure maintenance and improvement in quality of life among people living with MS are critical elements in the clinical management of this disease.

## 8. Role of Social and Environmental Determinants of Health

Beyond individual behaviors, social and environmental determinants play a critical role in shaping lifestyle choices, access to care, and disease outcomes in people with multiple sclerosis [36,37,38,39]. Socioeconomic status, education level, employment conditions, housing stability, social support networks, and access to green spaces or rehabilitation facilities may substantially influence the adoption and maintenance of healthy behaviors such as physical activity, balanced nutrition, and smoking cessation [36,37,38,39,40,41].

In MS, social disadvantage has been associated with higher disability levels, reduced quality of life, greater comorbidity burden, and lower adherence to both pharmacological and non-pharmacological interventions [37,41,42]. Differences in healthcare access and rehabilitation availability also contribute to variability in long-term outcomes and patient-reported measures [38,42]. Environmental barriers may be particularly relevant for individuals with progressive disease or higher disability, amplifying health disparities over time [39,42].

These observations highlight the need to consider lifestyle interventions within a broader public health and policy framework [36,37,38,39,40]. Addressing social and environmental determinants requires multilevel strategies, including community-based programs, accessible rehabilitation services, workplace accommodations, and policies aimed at reducing structural barriers to healthy living [37,38,39,40,41]. Integrating social context into lifestyle counseling may enhance the feasibility, equity, and long-term effectiveness of non-pharmacological interventions in MS [37,41].

## 9. Conclusions and Future Directions

This narrative review provides an integrative and updated synthesis of the evidence linking modifiable lifestyle factors with multiple sclerosis risk, progression, and clinical outcomes (Table 1). Across domains—including obesity and diet, physical activity, sleep, smoking, and alcohol use—current data support a biologically plausible and clinically relevant contribution of lifestyle exposures to immune regulation, metabolic balance, vascular function, and neuro-inflammatory processes. A key conceptual insight emerging from this framework is that several lifestyle factors converge on shared pathways, with glymphatic function representing a promising but still evolving mechanistic model rather than an established therapeutic target. However, to enhance clinical applicability, lifestyle interventions can be categorized based on the strength of available evidence. Smoking cessation, regular physical activity, sleep optimization, and weight control are supported by robust and consistent data and should be integrated systematically into routine MS management. In contrast, specific dietary patterns, vitamin D or omega-3 supplementation, alcohol intake, and glymphatic-targeted approaches show promise but are characterized by heterogeneous or inconclusive interventional evidence; these should be presented as supportive strategies rather than disease-modifying interventions. These areas should therefore be presented to patients as supportive and individualized strategies rather than disease-modifying treatments per se.

Regarding lifestyle interventions and DMTs, to our knowledge, current data do not identify any negative interactions. On the contrary, observational studies and guideline reviews suggest that lifestyle modifications—including Mediterranean-style diet, regular physical activity, stress reduction, and sleep optimization—may provide additive benefits to DMTs by improving symptom burden, reducing comorbidities, and creating a biologically favorable environment for treatment response. However, direct trials evaluating lifestyle–DMT interaction effects are lacking, making this an important direction for future research.

Other important research gaps remain. First, most available data are observational, and issues of residual confounding and reverse causation persist, particularly in patients with established disability. Second, validated biomarkers—especially for glymphatic function and lifestyle-related neurobiological pathways—are currently lacking in MS. Third, there is limited evidence on effect modification by age at onset, sex, disease phenotype, and treatment status. Fourth, high-quality randomized controlled trials testing structured, multidomain lifestyle interventions are still scarce.

Distinguishing direct causal effects of lifestyle factors from associations driven by disease severity or duration represents the major methodological challenge in MS research. However, prospective longitudinal studies and investigations conducted in individuals with clinically isolated syndrome or early MS reduce the risk of reverse causation by ensuring that lifestyle exposures precede disability-related behavioral changes. Mendelian randomization analyses further support causal inference by using genetic instruments for modifiable exposures such as BMI or smoking, thereby avoiding confounding by disease stage or clinical status. In addition, statistical frameworks—such as adjustment for EDSS and disease duration, sensitivity analyses using lagged models, and marginal structural models—help separate the impact of disability from that of lifestyle behaviors. When available, randomized controlled trials provide the highest level of causal evidence. Finally, mechanistic and biomarker-based studies can identify biological pathways influenced by lifestyle factors independently of disease progression, further strengthening causal interpretation.

In light of these conclusions, future translational and clinical research should prioritize the following: (1) longitudinal and genetically informed studies to strengthen causal inference; (2) development and validation of imaging and fluid biomarkers linked to metabolic and glymphatic mechanisms; (3) randomized trials combining disease-modifying therapies with targeted lifestyle interventions; and (4) pragmatic care models that integrate lifestyle assessment, behavioral counseling, and digital monitoring into multidisciplinary MS services.

Overall, embedding lifestyle medicine into MS prevention and care represents a feasible and potentially high-impact strategy. A structured, evidence-informed approach to lifestyle assessment and intervention—aligned with pharmacological treatment and social context—may improve long-term outcomes, reduce comorbidity burden, and enhance patient-centered care in multiple sclerosis.

## Figures and Tables

**Table 1:** Strength of evidence supporting lifestyle factors in multiple sclerosis.

Lifestyle Factor	Strength of Evidence	Type of Evidence	Clinical Recommendation	Notes
Smoking cessation	Strong	Consistent observational and interventional studies	Strongly recommended	Smoking accelerates progression; cessation slows disability worsening.
Physical activity	Strong	RCTs and observational studies	Recommended	Improves fatigue, mobility, QoL; may reduce relapse rates.
Weight control/obesity prevention	Strong	Mendelian randomization + epidemiological data	Recommended	Adolescent obesity increases MS risk through inflammatory mechanisms.
Sleep optimization	Moderate–Strong	Epidemiology, mechanistic, clinical associations	Recommended	Strong biological plausibility (glymphatic function); high symptom impact.
Mediterranean-style diet	Moderate	Observational studies; mechanistic support	Reasonable to recommend	Supports metabolic health; interventional evidence limited.
Specific dietary regimens	Weak–Moderate	Small heterogeneous trials	Optional; individualized	No consistent disease-modifying effects.
Vitamin D supplementation	Weak–Moderate	Observational associations, neutral RCTs	Ensure sufficiency only	Correct deficiency; avoid as stand-alone disease-modifying therapy.
Omega-3 and other supplements	Weak	Mixed or null RCTs	Not routinely recommended	Limited or inconsistent benefit.
Alcohol consumption	Weak/Mixed	Mechanistic data; limited clinical relevance	Advise moderation	High doses impair glymphatic/sleep function; low-dose effects uncertain.
Glymphatic-targeted lifestyle interventions	Emerging	Mechanistic + early translational	Not yet actionable	Promising but insufficient MS-specific evidence.

## Data Availability

No new data were created or analyzed in this study.
